# Sociodemographic, occupational, and personal factors associated with sleep quality among Chinese medical staff: A web-based cross-sectional study

**DOI:** 10.3389/fpubh.2022.1060345

**Published:** 2022-12-22

**Authors:** Yusheng Tian, Yuchen Yue, Jiaxin Yang, Hui Chen, Jizhi Wang, Junyu Liu, Hui Ding, Lulu Lu, Jiansong Zhou, Yamin Li

**Affiliations:** ^1^Department of Psychiatry, National Clinical Research Center for Mental Disorders, The Second Xiangya Hospital of Central South University, Changsha, China; ^2^Clinical Nursing Teaching and Research Section, The Second Xiangya Hospital, Central South University, Changsha, China; ^3^Department of Psychiatry, Restigouche Hospital Center, Campbellton, NB, Canada

**Keywords:** sleep quality, medical staff, associated factors, napping, bedtime phone use

## Abstract

**Background:**

Sleep quality among medical staff affects not only their own health but also the health of their patients. This study aimed to investigate the sociodemographic, occupational, and personal factors associated with sleep quality among medical staff in mainland China.

**Methods:**

An online survey was conducted from January 10 to February 5, 2019, involving 3,684 medical staff (female: 84.9%; mean age: 31.6 ± 7.7; age range: 18–72). Sleep quality was measured by the Chinese version of the Pittsburgh Sleep Quality Index (C-PSQI). Sociodemographic, occupational characteristics, and personal lifestyle factors were measured by standard questions. Binary logistic regression analyses were used to determine the factors associated with sleep quality.

**Results:**

57.9% (95% CI: 56.3–59.5%) of the study population experienced poor sleep quality (C-PSQI > 5). Binary logistic regression showed that poor sleep quality were associated with lower level of education, higher level of hospital care, longer weekly working hours, more than 30 min of cell phone use at bedtime, shift work (OR 1.33, 95% CI[1.12–1.58], *P* = 0.001), lack of regular naps (OR 1.46, 95% CI[1.26–1.69], *P* < 0.001) and lack of routine exercise (OR 1.69, 95% CI[1.46–1.97], *P* < 0.001).

**Conclusions:**

Poor sleep quality is highly prevalent among medical staff in mainland China. The findings indicate that appropriate strategies, such as implementing regular breaks, regulating overtime work and vacation interruptions, as well as developing exercise programs, relaxation training, and stress-management programs could help improve the sleep quality of medical staff.

## 1. Introduction

Sleep is an important and necessary part of our life. Feeling well rested and getting good quality sleep are crucial to human productivity and health (1). Poor sleep quality can have serious consequences, such as memory loss, impaired cognitive processing speed, as well as an increased risk of depression and anxiety disorders (2). Poor sleep quality has also been found to be associated with work-related injuries or accidents (3). In addition, people with sleep disorders report impaired quality of life (4, 5) and are at a higher risk of all-cause mortality (6). Sleep-disordered symptoms are highly prevalent all over the world, with the prevalence of self-reported insomnia ranging from 12.2 to 51.0% (7–9).

Medical staff has been reported to suffer from poor sleep due to high workload and night shift, especially in large-scale hospitals (10, 11). According to previous reports, sleep problems were four times more prevalent among medical staff than among the general population (11, 12). Studies have shown that sleep problems among healthcare professionals not only influence their own mental and physical wellbeing, but also the health of their patients and the quality of care they provide (13).

Several studies have reported sociodemographic and occupational factors associated with sleep quality among medical staff, but the findings have been inconsistent. Kunzweiler et al. (1) found that nurses older than 50 years experienced poor sleep quality more frequently than their younger colleagues, while Alboghdadly et al. (14) reported that poor sleep quality did not vary between age groups. In addition, Yu et al. (15) found that medical staff with junior professional titles were more likely to have poor sleep quality, on the contrary, Hsieh et al. (16) reported that professional title was not significantly associated with sleep quality among nurses. Marital status is also a factor related to sleep quality and it has been shown that people who are either married or divorced have poorer sleep quality than those who are not married (17). Occupational factors including shift work, work department, professional title and workload have been found to be associated with sleep quality among medical staff.

The association between sleep quality among medical staff and personal habits such as exercise, napping and cell phone use at bedtime has only been explored in a few studies to date. One of these studies showed that exercise was effective in improving sleep quality (18). Another recent study revealed that sleep quality was related to napping habit (19). Siesta, also known as afternoon sleep or midday nap is a common practice in some Asian countries. A few studies found that daytime naps were likely to disrupt nocturnal sleep and lead to poor sleep quality (20) while others reported that habitual napping could improve sleep quality (21, 22). With the widespread use of smartphones, many people have developed the habit of using their phones near bedtime; however, studies have shown that over-use of mobile phone near bedtime is one of the negative factors that can influence sleep quality and increase the risk of daytime cognitive impairments (23).

In mainland China, the workload of medical staff is very high. According to the 2018 national health yearbook, 8,180 million outpatients and 240 million inpatients received services provided by only 6.2 million doctors and 3.8 million nurses in the year 2017 (24). The high workload, tense doctor-patient relationship and night shift make Chinese medical staff vulnerable to poor sleep quality and require more attention (25). However, little information is available about sleep quality and its associated factors of medical staff in Mainland China. What's more, previous studies only included medical staff of one-level hospitals and many factors mentioned above were not collected and evaluated (26–28).

Therefore, this study aimed to investigate the sleep quality among medical staff in mainland China and to explore its associated factors including sociodemographic, occupational, and personal factors (exercise, napping and smartphone use at bedtime). To provide information for scientific management and improvement of sleep quality.

## 2. Materials and methods

### 2.1. Study design and population

This was a cross-sectional online survey that investigated the sleep quality among 3,684 front-line medical staff working in different departments across various levels of hospital care in mainland China. The survey was conducted from January 10 to February 5, 2019. The inclusion criteria were as follows: (i) at least 6 months experience working in a hospital, (ii) having the ability to read Chinese (as the survey was written in Chinese), and (iii) being willing to take part in the survey. The exclusion criteria were being under 18 years old and not completing the questionnaire entirely.

### 2.2. Survey procedures

This online survey was conducted using a web-based questionnaire (www.wjx.cn) through snowball convenience sampling. 40 nurses and doctors in the Second Xiangya Hospital of Central South University were initially invited to participate the survey and selected as “original deliverers.” They were asked to send the questionnaire links to their friends working in hospitals to participate in the online survey. And the link to the questionnaire was encouraged to spread among the respondents' WeChat (a Chinese social media APP) groups.

### 2.3. Ethical considerations

This study was conducted in accordance with the Helsinki Declaration and Institutional Research Ethics guidelines. This study was approved by the ethics committee of the National Clinical Research Center of the Second Xiangya Hospital (No. 2018S007). All respondents were informed about the objectives and aims of this study and e-informed consent was obtained before the survey.

### 2.4. Measures

#### 2.4.1. Sociodemographic characteristics and occupational factors

Sociodemographic information, including gender, age, marital status, and level of education (associate degree or below, bachelor's degree, and master's degree or above), as well as occupational information, including the level of hospital care (primary, secondary, and tertiary), professional titles (junior, mid-level, and senior), work schedule (shift or non-shift work), weekly working hours and years of experience were collected using a self-designed questionnaire.

#### 2.4.2. Personal habit factors

Three aspects of habits were measured, including napping, exercise, and smartphone use at bedtime. The napping habit was assessed by the question: Do you have the habit of having a lunch break or midday nap (Twice per week at least)? (29). Exercise habit was evaluated *via* the question: Do you have the habit of exercising (At least once per week, more than 30 min each time)? (30). Both questions were defined as dichotomous questions (Yes/No). The assessment of smartphone use at bedtime was based on the following question: How much time do you usually spend on your smartphone before going to bed? The multiple-choice answers included: Zero, 0–30, 30–60, 60–120, and more than 120 min.

#### 2.4.3. Sleep quality

Sleep quality was assessed using the Chinese version of the Pittsburgh Sleep Quality Index (C-PSQI, Cronbach's α = 0.77) (31). The C-PSQI consists of 18 items to evaluate seven aspects of sleep in the previous month, including the subject's sleep quality, sleep duration, sleep latency, sleep efficiency, sleep disturbances, use of sleep medication, and daytime dysfunction (32). The score of each component ranges from 0 to 3; the total score is the sum of the component scores and ranges from 0 to 21, with higher scores representing lower sleep quality. Study participants with a C-PSQI score >5 were classified as “poor sleep quality” (26).

### 2.5. Statistical analysis

All statistical analyses were performed using IBM software SPSS V.21.0 for Windows. Differences between good and poor sleep quality were examined by single-factor chi-square test. Odds ratios (ORs) and 95% confidence intervals (95% CI) were calculated to examine the association of sleep quality with occupational, personal, and demographic factors using binary logistic regression analysis (Forward LR). Initially, univariate analyses were performed, with each of the potential explanatory variables as independent variables, and sleep quality as the dependent variable. Pre-selection for the entry of all associated factors into the binary logistic regression model required a *P*-value of < 0.05 in the univariate analyses. A 5% significance level was accepted for all tests.

## 3. Results

Initially, a total of 3,706 respondents took part in the survey without any financial compensation. After removing 22 incomplete surveys, 3,684 respondents were included in the final analysis. 556 of these professionals were males (15.09%) and 3,128 were females (84.91%). The average age of the participants was 31.6 ± 7.7 years and the average C-PSQI score was 8.49 ± 3.49. Poor sleep quality (C-PSQI > 5) was recorded in 2,132 participants, accounting for 57.9% of the study population.

Table 1 shows that sleep quality was statistically related to age, level of education, level of hospital care, professional title, work schedule, weekly working hours, years of experience, profession, napping habit, exercise habit and smartphone use at bedtime (*P* < 0.05). Both gender and marital status were not statistically related to sleep quality in medical staff (*P* > 0.05).

**Table 1:** Distribution of good and poor sleep quality by sociodemographic, occupational, and personal variables.

**Variables**	**Sample size (*n* = 3,684)**	**With good sleep quality (*n* = 1,552)**	**With poor sleep quality (*n* = 2,132)**	**Statistics**	
	***N*** **(%)**	***N*** **(%)**	***N*** **(%)**	χ^2^	***P***
**Gender**					
Male	556 (15.1)	1,299 (41.5)	1,829 (58.5)		
Female	3,128 (84.9)	253 (45.5)	303 (54.5)	3.060	0.085
**Age group (years)**					
< 30	1,780 (48.3)	731 (41.1)	1,049 (58.9)		
30–39	1,306 (35.5)	536 (41.0)	770 (58.9)		
≥40	598 (16.2)	285 (47.7)	313 (52.3)	8.957	0.011
**Marital status**					
Married	2,423 (65.8)	1,027 (42.4)	1,396 (57.6)		
Unmarried	1,261 (34.2)	525 (41.6)	736 (58.4)	0.192	0.673
**Level of education**					
Associate degree or below	786 (21.3)	310 (39.4)	476 (60.6)		
Bachelor's degree	2,461 (66.8)	1,006 (40.9)	1,455 (59.1)		
Master's degree or above	437 (11.9)	236 (54.0)	201 (46.0)	29.190	< 0.001
**Level of hospital care**					
Tertiary hospital	2,541 (68.9)	1,063 (41.8)	1,478 (58.2)		
Secondary hospital	987 (26.8)	389 (39.4)	598 (60.6)		
Primary/community hospital	156 (4.2)	100 (64.1)	56 (35.9)	33.973	< 0.001
**Professional title**					
Senior	394 (10.7)	191 (48.5)	203 (51.5)		
Mid-level	1,021 (27.7)	459 (44.9)	562 (55.0)		
Junior	2,269 (61.6)	902 (39.8)	1,637 (60.3)	15.112	0.001
**Work schedule**					
Non-shift	984 (26.7)	505 (51.3)	479 (48.7)		
Shift	2,700 (73.3)	1,047 (38.8)	1,653 (61.2)	46.540	< 0.001
**Weekly working hours**					
>50 h	532 (14.4)	190 (35.7)	342 (64.3)		
40–50 h	2,140 (58.1)	885 (41.4)	1,255 (58.6)		
< 40 h	1,012 (27.5)	477 (47.1)	535 (52.9)	19.904	< 0.001
**Years of experience**					
< 5	1,382 (37.5)	588 (42.6)	794 (57.5)		
6–10	1,054 (28.6)	399 (37.9)	655 (62.1)		
11–20	766 (20.8)	326 (42.6)	440 (57.4)		
>20	482 (13.1)	239 (49.6)	243 (50.4)	19.042	< 0.001
**Profession**					
Nurse	2,750 (74.7)	1,119 (40.7)	1,631 (59.3)		
Physician	934 (25.3)	433 (46.4)	501 (53.6)	9.190	0.003
**Exercise habit**					
No	2,546 (69.1)	946 (37.2)	1,600 (62.8)		
Yes	1,138 (30.9)	606 (53.3)	532 (46.7)	83.565	< 0.001
**Regular napping**					
Yes	2,222 (60.3)	1,049 (47.2)	1,173 (52.8)		
No	1,462 (39.7)	503 (34.4)	959 (65.6)	59.303	< 0.001
**Bedtime phone use time**					
0	174 (4.7)	90 (51.7)	84 (48.3)		
< 30 min	1,731 (46.9)	805 (46.5)	926 (53.5)		
30–60 min	949 (25.8)	373 (39.3)	576 (60.7)		
60–120 min	487 (13.2)	165 (33.9)	322 (66.1)		
>120 min	343 (9.3)	119 (34.7)	224 (65.3)	44.638	< 0.001

Table 2 shows that sleep quality was independently related with age (30–39 years old, OR = 1.22, 95% CI [1.01–1.43]; ≥40 years old, OR = 1.36, 95% CI [1.09–1.69]), level of education (associate degree or below, OR = 1.83, 95% CI [1.41–2.39]; bachelor's degree, OR = 1.66, 95% CI [1.34–2.06]), level of hospital care (secondary hospital, OR = 2.27, 95% CI [1.57–3.23]; tertiary hospital, OR = 2.44, 95% CI [1.71–3.49]), weekly working hours: (40–50 h/week, OR = 1.24, 95% CI [1.06–1.45]; >50 h/week, OR = 1.59, 95% CI [1.26–1.99]), smartphone use at bedtime (30–60 min, OR = 1.48, 95% CI [1.06–2.09]; 60–120 min, OR = 1.83, 95% CI [1.26–2.64], >120 min, OR = 1.75, 95% CI [1.18–2.59]), shift work (OR = 1.33, 95% CI [1.12–1.58]), lack of regular napping (OR = 1.46, 95% CI [1.26–1.69]), and absence of exercise habit (OR = 1.69, 95% CI [1.46–1.97]). In other words, the risk factors for poor sleep quality in medical staff were a lower level of education, higher level of hospital care, longer weekly working hours, shift work, lack of regular napping, lack of physical exercise and more than 30 min of smartphone use at bedtime.

**Table 2:** Binary logistic regression analysis of factors associated with poor sleep quality among medical staff.

**The factors**		***B***	**SE**	***Wald***	***P***	**OR (95% CI)**
Age group	<30			9.801		Ref
	30–39	0.199	0.080	6.148	0.013	1.22 (1.04–1.43)
	≥40	0.306	0.112	7.409	0.006	1.36 (1.09–1.69)
Level of education	Master's degree or above			23.813		Ref
	Associate degree or below	0.607	0.135	20.340	<0.001	1.83 (1.41–2.39)
	Bachelor's degree	0.505	0.110	21.017	<0.001	1.66 (1.34–2.06)
Level of hospital care	Primary/community hospital			24.020		Ref
	Secondary hospital	0.819	0.186	19.321	<0.001	2.27 (1.57–3.23)
	Tertiary hospital	0.894	0.182	24.019	<0.001	2.44 (1.71–3.49)
Weekly working hours	<40 h			16.856		Ref
	40–50 h	0.217	0.079	7.468	0.006	1.24 (1.06–1.45)
	>50 h	0.462	0.116	15.853	<0.001	1.59 (1.26–1.99)
Bedtime phone use time	0			29.076		Ref
	<30 min	0.136	0.166	0.677	0.410	1.15 (0.83–1.59)
	30–60 min	0.395	0.173	5.193	0.022	1.48 (1.06–2.09)
	60–120 min	0.602	0.187	10.336	0.001	1.83 (1.26–2.64)
	>120 min	0.559	0.199	7.880	0.005	1.75 (1.18–2.59)
Shift work		0.282	0.087	10.466	0.001	1.33 (1.12–1.58)
Lack of routine exercise		0.527	0.077	46.463	<0.001	1.69 (1.46–1.97)
Lack of regular napping		0.377	0.074	25.815	<0.001	1.46 (1.26–1.69)

B, regression coefficient; SE, standard errors of regression coefficient.

The modified Hosmer-Lemeshow goodness-of-fit χ^2^ test statistics was 2.80 (*P* = 0.946).

## 4. Discussion

Our finding that 57.9% of the participants reported poor sleep quality is broadly consistent with the pooled prevalence (61.0%) of poor sleep quality among Chinese medical staff reported in a previous Meta-analysis of 53 studies (11).

Our study revealed that medical staff below 40 years of age and those without a master's degree or above suffered more frequently from poor sleep quality. These results were consistent with the findings of previous studies (33, 34). Considering this finding, it could be inferred that medical staff in mainland China with lower levels of education and younger ages are more likely to take up additional clinical work and night shifts in order to gain experience and refine the skill sets required in their specialties. In addition, they experience higher levels of pressure in the context of application for professional promotion, which makes them more vulnerable to sleep disturbances. Therefore, their sleep quality and psychological health issues deserve more attention.

Occupational factors including higher levels of hospital care, longer weekly working hours and shift work, which all reflect a heavy workload, were found to be significantly associated with poor sleep quality. Most patients treated in tertiary hospitals in China suffer from critical or serious illnesses or undergo major surgeries. Therefore, for medical staff in tertiary hospitals, staying up-to-date on advanced medical knowledge and clinical skills are essential, which may result in higher level of stress and lead to poor sleep quality (16). Various studies in the past have reported the association between shift work and negative health consequences such as heightened fatigue, anxiety, and poor sleep quality (16, 35, 36). Although shift work is unavoidable for medical staff, some measures could be taken on an individual and institutional level to improve sleep quality.

Maintaining good personal habits and leading a healthy lifestyle help to improve sleep quality and general wellbeing. Our study revealed that personal habits including napping, exercise and smartphone use at bedtime were significant associated factors for sleep quality among medical staff. Several studies have shown that a nap of < 30 min during lunch break could restore wakefulness and promote work performance (21, 37). In their study, Zhan et al. (33) found that frontline nurses without the habit of napping were more vulnerable to insomnia. However, in China, doctors and nurses are busy dealing with routine clinical work during the daytime and even too busy to have lunch, let alone a nap. Therefore, hospitals could set up strategies such as the implementation of regular mandatory breaks for clinical doctors and nurses as a short nap is helpful for regaining energy as well as improving sleep quality and work performance.

Physical exercise, an economical and non-pharmacological intervention, which is available to a vast majority of people, was found to be an effective approach for improving sleep quality (18, 38). This finding of our study was consistent with previous studies. However, no more than one-third of participants in this study were found to have regular exercise habit. Nurses and doctors as healthcare providers are expected to care more about their own health, and lead a healthy lifestyle; however, the low rate of exercise habit may result from a busy work schedule and shortage of fitness equipment in the hospital setting. Labor unions of hospitals can help overcome this challenge by advocating for more workout spaces, organizing regular physical activities, and setting up sports curriculums for healthcare workers. Moreover, medical staff should develop the habit of doing regular exercise to improve their own sleep quality and act as an example for their patients.

In our study, more than 30 min of smartphone use at bedtime was associated with poor sleep quality. Liese et al. (39) also found that bedtime mobile phone use predicted a delayed rise time, increased fatigue and higher insomnia score. Three mechanisms could explain how smartphone use at bedtime might influence sleep quality. First, the secretion of melatonin will be suppressed by the light from cell phone screens, delaying sleep onset, and disrupting the circadian rhythm (40, 41). Second, smartphone use at bedtime is an unstructured leisure activity and has no fixed start or endpoint, especially when done later in the day or as part of a bedtime routine, which can lead to symptoms of circadian rhythm sleep-wake disorders (42). Third, exposure to sexual or violent content in media may cause fright, arousal, or stress reactions (43). This stimulation may be associated with difficulties in falling asleep or poor sleep quality. A randomized pilot trial found that restricting phone use at bedtime was effective in reducing sleep latency, increasing sleep duration, reducing pre-sleep arousal and improving positive affect and working memory (44). Therefore, healthcare professionals should try to restrict bedtime phone use to improve their sleep quality.

### 4.1. Limitations

There are some limitations to this study. First, this study includes the use of cross-sectional data and self-report measures. Although there are theoretically sound reasons to assume that the above-mentioned factors could affect the sleep quality of medical staff, no solid conclusion regarding causal relationships can be made from the data derived from this cross-sectional study. Second, this study was an online cross-sectional survey, although 3,684 medical staff responded, the sample was small compared to the large population of medical staff in mainland China. Therefore, the sample has limited representativeness, and the prevalence of poor sleep quality measured in this study cannot fully represent the current status of sleep quality among Chinese medical staff.

## 5. Conclusion

This study shows that the overall rate of poor sleep quality among medical staff in mainland China is high. The associated factors for poor sleep quality were a lower level of education, higher level of hospital care, longer weekly working hours, shift work, lack of regular napping or routine exercise and more than 30 min of smartphone use at bedtime. A change in hospital policies coupled with individual efforts are crucial to improve sleep quality among medical staff through the management of the associated risk factors. Our study indicates that strategies such as the implementation of regular break times, the reduction of excessive overtime work, the regulation of vacation interruptions, and the establishment of exercise programs as well as relaxation and stress-management training could be developed to improve sleep quality and promote overall wellbeing among medical staff. In addition, medical staff themselves are expected to maintain healthy lifestyle habits. Sleep quality should be included on the list of objectives of health promotion as it is an important contributor to both the general health and workplace performance of medical staff.

## Data availability statement

The raw data supporting the conclusions of this article will be made available by the authors, without undue reservation.

## Ethics statement

The studies involving human participants were reviewed and approved by the Ethics Committee of the National Clinical Research Center of the Second Xiangya Hospital. Written informed consent for participation was not required for this study in accordance with the national legislation and the institutional requirements.

## Author contributions

JZ and YL contributed to conceiving the study concept and design. HC and YT analyzed the data and wrote the first draft of the paper. YY, JY, JW, LL, JL, and HD contributed to the interpretation of data and were involved in revising the manuscript for important intellectual content. All authors contributed to the article and approved the submitted version.

## References

[1] Kunzweiler K Voigt K Kugler J Hirsch K Bergmann A Riemenschneider H. Factors influencing sleep quality among nursing staff: results of a cross sectional study. Appl Nurs Res. (2016) 32:241–44. 10.1016/j.apnr.2016.08.007 27969035

[2] Dong H Zhang Q Sun Z Sang F Xu Y. Sleep disturbances among Chinese clinical nurses in general hospitals and its influencing factors. BMC Psychiatry. (2017) 17:241. 10.1186/s12888-017-1402-3 28673267 PMC5496307

[3] Shahly V Berglund PA Coulouvrat C Fitzgerald T Hajak G Roth T. The associations of insomnia with costly workplace accidents and errors: results from the America Insomnia Survey. Arch General Psychiatry. (2012) 69:1054–63. 10.1001/archgenpsychiatry.2011.2188 23026955

[4] Liu S Chow IHI Lu L Ren YM Yang HL Jian SY Ng CH Ungvari GS Wang F Xiang YT. Comparison of sleep disturbances between older nursing home residents in high- and low-altitude areas. J Geriat Psychiatry Neurol. (2020) 33:370–76. 10.1177/0891988719892335 31838930

[5] Chiu HFK Xiang Y-T Dai J Chan SSM Yu X Ungvari GS. Sleep duration and quality of life in young rural Chinese residents. Behav Sleep Med. (2013) 11:360–8. 10.1080/15402002.2013.764524 23461412

[6] Yin J Jin X Shan Z Li S Huang H Li P. Relationship of sleep duration with all-cause mortality and cardiovascular events: a systematic review and dose-response meta-analysis of prospective cohort studies. J Am Heart Assoc. (2017) 6:5947. 10.1161/JAHA.117.005947 28889101 PMC5634263

[7] Benbir G Demir AU Aksu M Ardic S Firat H Itil O. Prevalence of insomnia and its clinical correlates in a general population in Turkey. Psychiatry Clin Neurosci. (2015) 69:543–52. 10.1111/pcn.12252 25384688

[8] Nowicki Z Grabowski K Cubała WJ Nowicka-Sauer K Zdrojewski T Rutkowski M. Prevalence of self-reported insomnia in general population of Poland. Psychiatr Pol. (2016) 50:165–73. 10.12740/PP/58771 27086336

[9] Vaag J Saksvik-Lehouillier I Bjørngaard JH Bjerkeset O. Sleep difficulties and insomnia symptoms in Norwegian musicians compared to the general population and workforce. Behav Sleep Med. (2016) 14:325–42. 10.1080/15402002.2015.1007991 26337077

[10] Dong H Zhang Q Zhu C Lv Q. Sleep quality of nurses in the emergency department of public hospitals in China and its influencing factors: a cross-sectional study. Health Qual Life Outcomes. (2020) 18:116. 10.1186/s12955-020-01374-4 32349759 PMC7191763

[11] Zeng L-N Yang Y Wang C Li X-H Xiang Y-F Hall BJ. Prevalence of poor sleep quality in nursing staff: a meta-analysis of observational studies. Behav Sleep Med. (2020) 18:746–59. 10.1080/15402002.2019.1677233 31672062

[12] Cao X-L Wang S-B Zhong B-L Zhang L Ungvari GS Ng CH. The prevalence of insomnia in the general population in China: a meta-analysis. PLoS ONE. (2017) 12:e0170772. 10.1371/journal.pone.0170772 28234940 PMC5325204

[13] Feleke SA Mulatu MA Yesmaw YS. Medication administration error: magnitude and associated factors among nurses in Ethiopia. BMC Nurs. (2015) 14:53. 10.1186/s12912-015-0099-1 26500449 PMC4618536

[14] Alboghdadly A Saadh MJ Kharshid AM Shaalan MS Alshawwa SZ. Assessment of anxiety level and sleep quality of medical staff treating patients with COVID-19. Eur Rev Med Pharmacol Sci. (2022) 26:312–19. 10.26355/eurrev_202201_27783 35049010

[15] Yu SJ Cao YJ Ma DD. [A survey on night sleep quality and daytime tiredness among shift nurses in a tertiary teaching hospital]. Zhonghua lao dong wei sheng zhi ye bing za zhi = *Zhonghua laodong weisheng zhiyebing zazhi* = *Chin J Ind Hyg Occup Dis*. (2018) 36:855–58. 10.3760/cma.j.issn.1001-9391.2018.11.016 30646654

[16] Hsieh M-L Li Y-M Chang E-T Lai H-L Wang W-H Wang S-C. Sleep disorder in Taiwanese nurses: a random sample survey. Nurs Health Sci. (2011) 13:468–74. 10.1111/j.1442-2018.2011.00641.x 22011090

[17] Ouyang YQ Zhou WB Xiong ZF Wang R Redding SR. A web-based survey of marital quality and job satisfaction among Chinese nurses. Asian Nurs Res. (2019) 13:216–20. 10.1016/j.anr.2019.07.001 31323327

[18] Kelley GA Kelley KS. Exercise and sleep: a systematic review of previous meta-analyses. J Evid Based Med. (2017) 10:26–36. 10.1111/jebm.12236 28276627 PMC5527334

[19] Monk TH Buysse DJ Carrier J Billy BD Rose LR. Effects of afternoon “siesta” naps on sleep, alertness, performance, and circadian rhythms in the elderly. Sleep. (2001) 24:680–7. 10.1093/sleep/24.6.680 11560181

[20] Lin JN. Correlates and influences of taking an afternoon nap on nocturnal sleep in Chinese elderly: a qualitative study. Geriatr Nurs. (2018) 39:543–47. 10.1016/j.gerinurse.2018.03.002 29653772

[21] Katagi A Miyai N. [Effects of short-term nap and light physical exercise on sleep among elderly patients with mild-to-moderate dementia in communal living group homes]. Nihon eiseigaku zasshi Jpn J Hyg. (2018) 73:365–72. 10.1265/jjh.73.365 30270304

[22] McDevitt EA Sattari N Duggan KA Cellini N Whitehurst LN Perera C. The impact of frequent napping and nap practice on sleep-dependent memory in humans. Sci Rep. (2018) 8:15053. 10.1038/s41598-018-33209-0 30305652 PMC6180010

[23] Carter B Rees P Hale L Bhattacharjee D Paradkar MS. Association Between Portable Screen-Based Media Device Access or Use and Sleep Outcomes: A Systematic Review and Meta-analysis. JAMA Pedia. (2016) 170:1202–08. 10.1001/jamapediatrics.2016.2341 27802500 PMC5380441

[24] Commission NH. China Health Statistics Year-Book. Beijing: Peking Union Medical College Press (2018).

[25] Ma J Chen X Zheng Q Zhang Y Ming Z Wang D. Serious workplace violence against healthcare providers in China between 2004 and 2018. Front Public Health. (2020) 8:574765. 10.3389/fpubh.2020.574765 33520908 PMC7841458

[26] Chang Q Xia Y Bai S Zhang X Liu Y Yao D. Association between Pittsburgh sleep quality index and depressive symptoms in Chinese resident physicians. Front Psychiatry. (2021) 12:564815. 10.3389/fpsyt.2021.564815 34149465 PMC8206480

[27] Deng X Liu X Fang R. Evaluation of the correlation between job stress and sleep quality in community nurses. Medicine. (2020) 99:e18822. 10.1097/MD.0000000000018822 31977875 PMC7004582

[28] Sun T Gao L Li F Shi Y Xie F Wang J. Workplace violence, psychological stress, sleep quality and subjective health in Chinese doctors: a large cross-sectional study. BMJ Open. (2017) 7:e017182. 10.1136/bmjopen-2017-017182 29222134 PMC5728267

[29] Mohammad Y. Siesta and risk for ischemic stroke: results from a case-control study. Medicina. (2020) 56:222. 10.3390/medicina56050222 32392748 PMC7279277

[30] Kong L Cui Y Gong Q. Duration of keeping an exercise habit and mental illness and life attitude among University students. Int J Environ Res Public Health. (2022) 19:669. 10.3390/ijerph191811669 36141940 PMC9517129

[31] Tsai P-S Wang S-Y Wang M-Y Su C-T Yang T-T Huang C-J. Psychometric evaluation of the Chinese version of the Pittsburgh Sleep Quality Index (CPSQI) in primary insomnia and control subjects. Qual Life Res. (2005) 14:1943–52. 10.1007/s11136-005-4346-x 16155782

[32] Buysse DJ Reynolds CF Monk TH Berman SR Kupfer DJ. The Pittsburgh Sleep Quality Index: a new instrument for psychiatric practice and research. Psychiatry Res. (1989) 28:193–213. 10.1016/0165-1781(89)90047-4 2748771

[33] Zhan Y Liu Y Liu H Li M Shen Y Gui L. Factors associated with insomnia among Chinese front-line nurses fighting against COVID-19 in Wuhan: a cross-sectional survey. J Nurs Manag. (2020) 28:1525–35. 10.1111/jonm.13094 32657449 PMC7405094

[34] Cui ZY Li L Wang Y Chao H Huang Z. [A survey of sleep quality of employed nurses in military hospitals]. Zhonghua lao Dong wei Sheng zhi ye Bing za zhi= *Zhonghua Laodong Weisheng Zhiyebing Zazhi*= *Chin J Ind Hyg Occup Dis*. (2017) 35:578–81. 10.3760/cma.j.issn.1001-9391.2017.08.005 29081124

[35] Tribis-Arrospe B Ballesteros-Peña S Abecia Inchaurregui LC Egea-Santaolalla C Guerra-Martin L de Larrinaga AÁ. [Sleep quality and adaptation to shift-work among ambulance staff in the Basque Country]. An Sist Sanit Navar. (2020) 43:189–202. 10.23938/ASSN.0871 32814924

[36] Pérez-Fuentes MDC Jurado MDMM Márquez MDMS Linares JJG. Analysis of sociodemographic and psychological variables involved in sleep quality in nurses. Int J Environ Res Public Health. (2019) 16:3846. 10.3390/ijerph16203846 31614622 PMC6843758

[37] Dhand R Sohal H. Good sleep, bad sleep! The role of daytime naps in healthy adults. Curr Opin Pulmon Med. (2006) 12:379–82. 10.1097/01.mcp.0000245703.92311.d0 17053484

[38] Yang P-Y Ho K-H Chen H-C Chien M-Y. Exercise training improves sleep quality in middle-aged and older adults with sleep problems: a systematic review. J Physiother. (2012) 58:157–63. 10.1016/S1836-9553(12)70106-6 22884182

[39] Exelmans L Van den Bulck JJS. Bedtime mobile phone use and sleep in adults. Soc Sci Med. (2016) 148:93–101. 10.1016/j.socscimed.2015.11.037 26688552

[40] Chellappa SL Steiner R Oelhafen P Lang D Götz T Krebs J. Acute exposure to evening blue-enriched light impacts on human sleep. J Sleep Res. (2013) 22:573–80. 10.1111/jsr.12050 23509952

[41] Wood B Rea MS Plitnick B Figueiro MG. Light level and duration of exposure determine the impact of self-luminous tablets on melatonin suppression. Appl Ergon. (2013) 44:237–40. 10.1016/j.apergo.2012.07.008 22850476

[42] Levenson JC Shensa A Sidani JE Colditz JB Primack BA. The association between social media use and sleep disturbance among young adults. Prevent Med. (2016) 85:36–41. 10.1016/j.ypmed.2016.01.001 26791323 PMC4857587

[43] Anderson CA Shibuya A Ihori N Swing EL Bushman BJ Sakamoto A. Violent video game effects on aggression, empathy, and prosocial behavior in eastern and western countries: a meta-analytic review. Psychol Bull. (2010) 136:151–73. 10.1037/a0018251 20192553

[44] Ye J Wen Y Chu X Li P Cheng B Cheng S. Association between herpes simplex virus 1 exposure and the risk of depression in UK Biobank. Clin Trans Med. (2020) 10:e108. 10.1002/ctm2.108 32564518 PMC7403656

